# Disparities in access to mobile devices and e-health literacy among breast cancer survivors

**DOI:** 10.1007/s00520-021-06407-2

**Published:** 2021-07-08

**Authors:** Zoe Moon, Mira Zuchowski, Rona Moss-Morris, Myra S. Hunter, Sam Norton, Lyndsay D. Hughes

**Affiliations:** Health Psychology Section, Institute of Psychiatry, Psychology & Neuroscience (IoPPN), King’s College London, 5th Floor Bermondsey Wing, Guy’s Hospital, London, SE1 9RT UK

**Keywords:** e-Health, Apps, Breast cancer, Survivorship, m-Health, Inequalities

## Abstract

**Background:**

The number of e-health interventions developed for breast cancer survivors continues to increase. However, issues with engagement and retention are common. This study aimed to explore e-health literacy rates and access to smartphones and tablets in a large sample of breast cancer survivors.

**Methods:**

In study 1, women were recruited from outpatient breast clinics across England and Wales. Eligible women were asked to complete a questionnaire pack to assess their access to devices and their e-health literacy. Multiple regression analyses were run to assess the relationship between technology access and e-health literacy with sociodemographic variables such as age, social deprivation, and education. Study 2 presents a smaller sample recruited through social media who answered a questionnaire relating to use of mobile devices and e-health, and apps.

**Results:**

Two thousand nine women participated in the study. Seventy-one percent had access to a smartphone, 54% had access to a tablet, and 20% did not have access to either device. Multiple logistic regressions showed that women who were younger, had higher levels of education, and who were from less deprived areas were more likely to have access to either device. Poorer e-health literacy was associated with being older, having less education, and not having access to a mobile device.

**Conclusions:**

Whilst the results show relatively widespread access to mobile devices, there is evidence of a digital divide across some groups. Online interventions should be developed with consideration of individuals who are less e-health-literate and less technologically adept in order to increase the likelihood of engagement.

**Supplementary Information:**

The online version contains supplementary material available at 10.1007/s00520-021-06407-2.

## Introduction

Over the past 5 years, interest in e-health and m-health interventions for people diagnosed with breast cancer has increased. For example, app-based programmes have been developed for pain management, mindfulness, symptom burden, and medication adherence [1–4]. Systematic reviews and meta-analyses have shown that these interventions can significantly improve fatigue, physical activity, and depression in cancer survivors [5–7]. There are considerable benefits to utilizing e-health interventions, including low cost, broad reach, and the potential for widespread implementation. However, issues with uptake and retention are common [8–10]. For example, in a trial of an online mindfulness-based cognitive therapy for anxiety and depression in cancer survivors, 80% of participants began treatment, but just over half (56%) completed all eight modules [11]. Dropout rates of 30–40% are common, with some interventions reporting attrition rates as high as 70% [8, 10, 12].

Low uptake with e-health applications may be due in part to the age of breast cancer survivors, who may be less likely to have access to the necessary devices. The highest incidence rates for breast cancer are found in women aged 65–69, and almost half of cases are in women over the age of 65 [13]. Furthermore, many of these e-health interventions are aimed at breast cancer survivors many years post diagnosis. Data from Great Britain shows that only 80% of those aged 65 or older have access to the Internet, and only 67% of retired adults report recent Internet use [14, 15]. Access to smartphones and tablets is also considerably lower in adults over the age of 65 [16]. These access issues are likely to have a substantial impact on the uptake of e-health interventions. Furthermore, studies have shown that older adults are less likely to use the Internet, to be users of mobile health apps, and to seek cancer information online [9, 17, 18]. This suggests there are also likely to be issues with long-term engagement in this age group, as they may be less familiar or comfortable with using e-health interventions or accessing information online.

As well as Internet access and familiarity with devices, engagement with e-health interventions is likely to be affected by health literacy, or more specifically, e-health literacy. Health literacy refers to the degree to which individuals have the capacity to obtain, process, and understand basic health information and services needed to make appropriate health decisions [19]. e-Health literacy relates specifically to the ability to find, understand, and appraise health information from electronic sources [20]. Studies in the USA have found that patients with lower health literacy were less likely to own and use a mobile phone for health-related purposes [17, 21].

There are concerns that these differences in access to and engagement with e-health interventions may be exacerbating existing social health inequalities such as those seen in cancer mortality rates [21, 22]. Research has shown that groups who are less likely to access or engage with e-health interventions may already be at risk of poorer health outcomes. For example, Internet access and health literacy are lower in those who are older, who have less formal education, and have lower socioeconomic status [23–26]. In a survey of 500 cancer survivors in the USA, participants who were unemployed and had lower levels of education were less likely to seek cancer information online [9]. Another large US survey found that cancer survivors who reported not using the Internet were more likely to belong to an ethnic minority, be less educated, and reside in rural areas [18].

Whilst the use of e-health to support women diagnosed with breast cancer continues to grow, little attention has been paid to the e-health literacy skills and technology access of these women. Gaining a greater understanding in these areas will provide important information on the potential scalability, reach, and efficacy of these interventions. The aims of this study were to explore e-health literacy and access to smartphones and tablets in a large sample of breast cancer survivors in the UK, and to explore health-related app usage in a smaller online sample. The specific aims of the research were to:

-
  - Identify what proportion of women diagnosed with primary breast cancer has access to smartphones and tablets.
  - Explore differences in access across the following demographic variables: age, ethnicity, years of education, rural/urban residence, and level of socioeconomic deprivation.
  - Identify the rates of e-health literacy and explore if e-health literacy differs across the following demographic variables: age, ethnicity, years of education, rural/urban residence, and level of socioeconomic deprivation.
  - Explore health-related app usage in a smaller sample of women with technology access.

## Methods

Two separate studies are described. Study 1 presents a large dataset recruited from NHS clinics. Study 2 presents a smaller sample of women who were recruited online.

### Study 1 (NHS clinic recruitment)

#### Procedure and participants

This study was a secondary analysis conducted as part of a larger longitudinal observational study of women with oestrogen receptor–positive (ER +) breast cancer prescribed hormonal therapy. The study received full ethical approval from London—City & East NHS Research Ethics Committee (18/LO/1674). Participants were recruited from outpatient breast cancer clinics across NHS Trusts in England and Wales. Participants were eligible for inclusion if they met the following criteria: (1) they were female; (2) aged over 18; (3) received a diagnosis of primary breast cancer; (4) prescribed hormone therapy within the last 3 years. Participants were excluded if they had secondary breast cancer or were not proficient in verbal and written English. A member of the research or clinical care team identified eligible participants and provided written and verbal information about the study. Written informed consent was obtained from interested participants who then completed a baseline questionnaire. The questionnaire could be completed on paper or via a secure online survey platform (Online Surveys, JISC).

#### Measures

*e-Health literacy* was assessed using the eHEALS scale [20]. This includes 8 items measured on a 5-point Likert scale, with scores ranging from 8 to 40 (higher scores indicate higher literacy). The scale has widespread use and demonstrates high internal consistency (α = 0.94) [27].

Access to a smartphone and/or tablet was self-reported based on the Oxford English Dictionary definitions of these devices. Participants also self-reported sociodemographic and clinical variables including age, ethnicity, years of full-time education, and previous treatment. Socioeconomic status was measured using the Index of Multiple Deprivation (IMD), a UK Government measure of relative neighbourhood deprivation, based across measures of income, employment, education, health and disability, crime, barriers to housing and services, and living environment [28]. IMD scores are ranked across 32,844 areas from most to least deprived. The IMD quintile is calculated by dividing the 32,844 ranks into five equal groups, from 1 (most deprived) to 5 (least deprived). The Rural/Urban Classification is a UK Government statistic used to distinguish between rural and urban areas based on the resident population.

#### Sample size

This study was powered as part of a larger observational study; therefore, there is no specific a priori sample size calculation for this analysis. However, a post hoc power calculation using G*Power 3.1.9.6 determined that with five predictor variables (age, IMD quintile, ethnicity, age left education, technology access), an effect size of *f*^2^ = 0.14, a sample size of *n* = 1870, and α = 0.05, the study has > 99% statistical power to detect the required effect [29].

### Study 2 (online recruitment)

#### Procedure and participants

The study was given institutional ethical approval (PNM Research Ethics Panel, LRS-17/18–5414). Participants were recruited through online advertisements on social media. Interested participants contacted the research team, where they were shown the Information Sheet for the study and were screened for eligibility using an online form. All participants were informed that completing questionnaires implied consent to participate. Eligible participants were female, in the UK, had been diagnosed with ER + breast cancer, and had been prescribed adjuvant hormone therapy. Participants were excluded if they had secondary breast cancer or were not proficient in verbal and written English. If eligible, participants completed an online questionnaire via a secure survey platform (Online Surveys, JISC).

#### Measures

Participants were asked to self-report sociodemographic and clinical variables (age, ethnicity, relationship status, job status, previous treatment, cancer stage). They then completed a series of questions relating to their current use of the Internet, devices, and apps, based on methodology from the Office of National Statistics (ONS). Participants were asked how often they used different devices, how often they used apps, what they currently used apps for, and whether they would want to access e-health interventions through apps, a website or both.

#### Sample size

There is no sample size calculation for study 2 as the purpose is descriptive, rather than testing statistically significant effects.

### Data analysis

Statistical analyses were conducted using IBM SPSS v26. For study 1, item level missing data on the eHEALS was < 1% and was replaced using mean substitution. Seven percent of participants (n = 139) did not complete the eHEALS and were therefore not included in the eHEALS analysis. Women who did not complete the eHEALs were older (72 vs. 60, *p* < 0.001), were younger when they left full-time education (16 vs. 18, *p* < 0.001), and were from a more deprived area (5.4 vs. 6.0, *p* = 0.009). Forty-nine percent of these indicated that they did not complete the scale as they did not have access to the Internet. Independent groups *t*-test and chi-squared tests were used to compare clinical and demographic characteristics across those who did and did not have access to either a smartphone or a tablet. A multivariate logistic regression was then carried out to identify factors associated with access to either device. One-way ANOVAs were used to compare scores on the eHEALS across different clinical and demographic variables. Factors which differed significantly were entered into a multivariate linear regression to predict eHEALS scores. Estimates for health-app usage and preference for future e-health intervention delivery for the combined sample were calculated by back-weighting data using the NHS clinical sample proportions (see Online Resource 1).

## Results

### Study 1 (NHS clinic recruitment)

Three thousand one hundred twenty women were invited to participate across 19 NHS Trusts in England and Wales. Two thousand nine (64%) agreed to participate in the study and completed the baseline questionnaire. Participant characteristics are shown in Table 1. The mean age was 60.5 (SD = 11.3, range 28–100). Ninety-two percent of women were White British, and 73% were from a rural area. Mean age left full-time education was 18 (SD = 4.3).

**Table 1:** Bivariate associations between technology access and participant characteristics (n = 2009)

	Total sample demographics n (%)	Smartphone access (*n* = 1416)	Tablet access (*n* = 1082)	Access to either smartphone or tablet (*n* = 1612)
Age		***	***	***
≤ 45	163 (8%)	97%	64%	97%
46–55	547 (28%)	86%	58%	90%
56–65	581 (29%)	75%	55%	84%
66–75	513 (26%)	55%	50%	71%
> 76	186 (9%)	31%	41%	53%
Age left full-time education		***	***	***
< 16	373 (19%)	44%	42%	62%
16	590 (29%)	72%	51%	80%
17	193 (10%)	71%	57%	82%
18	304 (15%)	81%	60%	89%
> 18	516 (26%)	82%	63%	90%
Ethnicity		***		**
White British	1845 (92%)	69%	54%	80%
Other ethnic groups	156 (8%)	86%	57%	89%
Rural Urban Classification				
Urban	1445 (73%)	71%	54%	81%
Rural	526 (27%)	68%	55%	79%
IMD quintile		***	***	***
1 (most deprived)	258 (13%)	60%	47%	72%
2	358 (18%)	68%	51%	79%
3	444 (23%)	73%	53%	82%
4	496 (25%)	71%	53%	80%
5 (least deprived)	416 (21%)	76%	63%	85%
Breast cancer stage				
Stage 1	792 (40%)			
Stage 2	855 (44%)			
Stage 3	224 (11%)			
Unsure	87 (4%)			
Time since diagnosis				
< 6 months	220 (12%)			
6–12 months	389 (20%)			
1–2 years	720 (38%)			
2–3 years	442 (23%)			
3–4 years	150 (8%)			

Independent groups t-test and chi-squared tests. *Statistically significant relationship at p < 0.05, **statistically significant relationship at p < 0.01, ***statistically significant relationship at p ≤ 0.001. *IMD*, Index of Multiple Deprivation

### Access to mobile devices

Across the whole dataset, 71% of women (*n* = 1416) reported having access to a smartphone. Only 54% reported having access to a tablet (*n* = 1082). Twenty percent (*n* = 393) did not have access to either a smartphone or a tablet. Bivariate analyses showed that women with access to any mobile device tended to be younger, to have received more years of full-time education, and be from a less deprived area (Table 2). Women who were White British were less likely to have access than women from other ethnic groups. There was no difference in access to these devices across the Rural/Urban Classification.

Multivariate logistic regression predicting access to any mobile device showed that age, education, and deprivation all had independent associations with access (Table 3). Women who were older had lower odds of having access to a device (OR = 0.93, CI = 0.91–0.94, *p* < 0.001). Women who had more years of full-time education had higher odds of having access to a device (OR = 1.10, 95% CI = 1.05–1.16,* p* < 0.001). Compared to women in the least deprived area, those in the two most deprived quintiles were up to 60% less likely to have access to a device, but access was still high overall, with over three-quarters of these women reporting access.

**Table 2:** Multivariate logistic regression predicting access to mobile devices (smartphone or tablet) (n = 2009)

	OR (95% CI)	*p*
Age	0.93 (0.91–0.94)	< 0.001
Age left full-time education	1.10 (1.04–1.16)	< 0.001
Ethnicity (White British)	0.88 (0.49–1.56)	0.674
IMD quintile Quintile 1 (most deprived) Quintile 2 Quintile 3 Quintile 4 Quintile 5 (least deprived)	Wald: 19.979 0.40 (0.26–0.61) 0.62 (0.41–0.94) 0.76 (0.51–1.13) 0.75 (0.51–1.10) Reference	0.001 < 0.001 0.023 0.178 0.139 Reference

Nagelkerke R^2^ = 0.195, chi-squared = 249.157, p < 0.001. *IMD*, Index of Multiple Deprivation.

### e-Health literacy

Scores on the eHEALS ranged from 8 to 40, with a mean score of 28.8 (*SD* = 7.34). Table 3 shows bivariate associations between e-health literacy and sociodemographic variables. Women who were younger and who had more years of full-time education had higher e-health literacy scores than women who were older and had less full-time education. Women who had access to any mobile device had higher e-health literacy scores than women without access. Women who were White British had lower e-health literacy than women from other ethnic groups. Deprivation level and Rural/Urban Classification were not significantly associated with e-health literacy.

A multiple linear regression analysis showed that age, access to mobile devices, education, and the 3rd IMD quintile had independent associations with e-health literacy (Table 4). For every decade increase in age, eHEALS score decreases by 1.7 units (95% CI = 0.20–0.14, *p* < 0.001). This is the most impactful factor in the model (standardized beta =  − 0.25). Individuals with access to any mobile device have a higher eHEALS score by 3.27 units (95% CI = 2.33–4.22, *p* < 0.001). For every additional year of full-time education, eHEALS score increases by 0.13 units (95% CI = 0.04–0.20, *p* = 0.003, standardized beta = 0.07). Relative to individuals in the 5th IMD quintile, the least deprived group, individuals in the 3rd IMD quintile have an eHEALS score lower by 1.02 units (95% CI = 1.99–0.05, p = 0.04). The model overall explains 12.3% of variability in e-health literacy. Post hoc power analysis indicated that the power to detect obtained effects at the α = 0.05 level was > 0.99 for the overall regression in predicting e-health literacy.

**Table 3:** e-Health literacy scores across different variables (n** = **1860)

	eHEALS scores mean (SD)	N	Association with e-health literacy
Age			***
≤ 45	31.38 (6.60)	161	
46–55	30.73 (5.98)	540	
56–65	29.32 (6.64)	561	
66–75	27.01 (7.98)	456	
> 76	22.85 (8.93)	127	
Age left full-time education			***
< 16	25.19 (8.53)	301	
16	28.55 (6.93)	555	
17	29.44 (6.34)	181	
18	29.91 (6.89)	300	
> 18	30.87 (6.82)	497	
Access to mobile devices			***
Yes	29.58 (6.83)	1572	
No	25.04 (8.72)	288	
Ethnicity			**
White British	28.73 (7.37)	1708	
Other ethnic groups	30.68 (6.81)	147	
Rural Urban Classification			-
Urban	28.88 (7.45)	1346	
Rural	28.89 (7.02)	479	
IMD quintile			-
1 (most deprived)	28.74 (7.00)	228	
2	28.72 (7.59)	336	
3	28.42 (7.83)	413	
4	29.01 (7.08)	351	
5 (least deprived)	29.44 (7.08)	398	

One-way ANOVAs. *Statistically significant relationship at p < 0.05, **statistically significant relationship at p < 0.01, ***statistically significant relationship at p < 0.001. *IMD*, Index of Multiple Deprivation

### Study 2 (online recruitment)

One hundred thirty-six women completed the online questionnaire. Mean age was 50 (SD = 8.0). All the women in the online study had access to either a smartphone or a tablet. Additionally, 114 (84%) also had access to a laptop or desktop computer. The majority of participants (94%) reported using an app daily or almost every day (Table 5). This was lower in the oldest group (88%) than the youngest group (97%). In terms of health-related apps, 49% reported using a health-related app in the last week, and 18% had never used one. The proportion of women who had never used a health-related app was significantly higher in the oldest group (31%) than in the younger two age groups.

**Table 4:** Multiple linear regression analysis predicting e-health literacy

	Unstandardized β *(SE)*	Standardized coefficient beta	95% CI
Age	− 0.17*** (0.02)	− 0.25	(− 0.20)–(− 0.14)
IMD quintile			
1 (most deprived)	− 0.50 (0.59)	− 0.02	(− 1.65)–(0.66)
2	− 0.67 (0.52)	− 0.04	(− 1.69)–(0.35)
3	− 1.02* (0.49)	− 0.06	(− 1.99)–(− 0.05)
4	− 0.34 (0.48)	− 0.02	(− 1.28)–(0.61)
5 (least deprived)	Reference	Reference	Reference
Ethnicity (White British)	0.02 (0.64)	0.001	(− 1.24)–(1.27)
Age left full-time education	0.13** (0.04)	0.07	(0.04)–(0.20)
Access to mobile devices	3.27*** (0.47)	0.16	(2.33)–(4.20)
* R*^2^		0.12	
* F*		31.06***	

*Statistically significant relationship at p < 0.05, **statistically significant relationship at p < 0.01, ***statistically significant relationship at p < 0.001. *IMD*, Index of Multiple Deprivation

**Table 5:** Reported app usage in the online sample

	Total sample	Aged < 45 (n = 32)	Aged 46–55 (n = 73)	Aged 56 + (n = 29)	
Device access					
Smartphone	96%	100%	96%	93%	0.354
Mobile phone	13%	13%	11%	14%	0.918
Tablet	74%	72%	71%	83%	0.469
Computer	84%	94%	78%	90%	0.085
App usage (general)					*p* < 0.001
More than once a day	83%	91%	84%	76%	
Daily or almost everyday	11%	6%	12%	14%	
Weekly or less	6%	3%	4%	7%	
Health-related app use					*p* = 0.011
Within the last week	49%	47%	52%	48%	
Longer than a week ago	33%	41%	33%	21%	
Never used	18%	13%	15%	31%	
Preference for support programme					*p* = 0.853
Online via a website	9%	6%	10%	14%	
An app for smartphones/tablets	33%	38%	32%	29%	
Both a website and an app	54%	53%	56%	50%	
Neither	4%	3%	3%	7%	
Current app usage					
Messaging and social	97%	100%	99%	90%	
Utilities and productivity	46%	50%	47%	41%	
Health	65%	67%	66%	59%	
Business and finance	28%	31%	32%	17%	
Shopping	67%	75%	66%	62%	
Entertainment	38%	47%	43%	17%	

When asked their preference for future support programmes, 9% of the sample reported a preference for an online only programme, although this was slightly higher in the oldest group (14%). Around a third of women across all ages reported a preference for an app-based intervention. Most women reported a preference for both a website and an app (54%) and this was similar across age groups.

Combining the proportions of both samples, across all ages, it is estimated that 18–34% would have never used a health app with estimates of 13–16% for under 45 s and 31–49% for 55 + . It is estimated that 4–23% of women across all ages would prefer neither online nor app-based intervention (see Online Resource 1).

## Discussion

Study 1 explored access to mobile devices and e-health literacy in a large sample of breast cancer survivors recruited from UK NHS clinics. Results showed that the majority (80%) of this population do report access to mobile devices, providing tentative support for e-health interventions. This was further supported by study 2 which showed that 94% of participants recruited online in the UK reported using mobile apps daily, and 49% reported using a health-related app in the past week. However, one-fifth of the NHS-recruited sample did not have access to either a smartphone or a tablet, meaning they may not be able to access or fully engage with mobile interventions. Women who were younger, who had more full-time education, and who were from less socioeconomically deprived areas were more likely to have access to these mobile devices.

As expected, older women were less likely to have access to mobile devices and had lower e-health literacy. Just under half of women over 65 did not have access to a smartphone; however, when looking across both devices, the percentage of women aged 65 + without access fell to only 29%. This suggests that these age disparities may not be as pronounced as previously thought, which is supported by evidence suggesting that this age gap has been closing over recent years [14]. Additionally, recent research has suggested that older adults might be more likely to engage with online interventions than younger adults [4, 30]. However, there is evidence to suggest that long-term digital engagement may still be lower in older adults [32]. Furthermore, the digital divide is much stronger in women aged over 75, where only 53% of women had access to either a smartphone or a tablet. This is supported by recent research highlighting that digital exclusion is a particular problem for those over 75 [33]. Consideration should be taken on how to develop interventions which are inclusive for this older cohort of women, who make up a quarter of all new breast cancer diagnoses [13]. However, it is important to note that due to a range of personal, social, and institutional factors, the exclusion of this group is a pervasive issue across all intervention types and is not specific to e-health interventions [34].

As well as age, access to mobile devices was also associated with years of full-time education and lower deprivation levels. This is consistent with previous literature showing that people from more deprived areas were less likely to own smartphones, and validates concerns that e-health interventions may disadvantage certain groups and exacerbate existing disparities [21, 22]. However, the relationship between deprivation and mobile device access does not appear to be linear, with the effects being most pronounced in the two most deprived quintiles. Compared to women in the least deprived area, those in the most deprived areas were around 60% less likely to have access to a device, although overall access was still high at around 75%. Consideration also needs to be given to barriers beyond device access, such as sharing devices with family members; limited space on devices; and having access to charge, credit, and repair facilities, which may further impact implementation and accessibility of e-health interventions [35, 36].

Interestingly, whilst mobile device access differed across both socioeconomic status and education levels, e-health literacy remained fairly constant and moderately high across the different deprivation levels. This contrasts with previous research showing a relationship between socioeconomic status and health literacy [25, 37, 38]. This may be due to the varied measurements of socioeconomic status used across studies. Neter and Brainin [23] found a significant relationship between e-health literacy and socioeconomic status when using education as a proxy for socioeconomic status. It is likely that it is the educational aspect of socioeconomic status which is most strongly related to e-health literacy. This could explain why the IMD, which provides a more nuanced measure of area deprivation, was not associated with e-health literacy in this study, whilst education was.

Results also showed that White women were less likely to have access to mobile devices, and had slightly lower e-health literacy, than women from other ethnic groups. This is unlikely to be associated with socioeconomic status, as IMD deprivation scores were similar across ethnic groups. However, it may be an artefact of age, as the White women in this sample were significantly older, and ethnicity was not a significant predictor of technology access or e-health literacy in the multivariate model when controlling for age. Whilst these results contrast with previous research in the USA [18, 39], it is in line with recent UK data showing that adults who are White had the lowest rate of recent Internet usage and were least likely to use a website or app to make a healthcare appointment [40, 41].

Overall, the results suggest that access to these devices was relatively widespread, which supports the use of e-health interventions in this group. However, it is important to acknowledge that access to these devices does not necessarily lead to uptake or sustained engagement with these interventions [32]. Furthermore, there is some evidence of a digital divide, and ways to overcome this and engaging those with lower health literacy need to be taken into consideration by future e-health interventions to increase inclusivity and avoid exacerbating existing health disparities. There is evidence that e-health training courses for older adults may be helpful [42]. However, there are also changes that can be made at the intervention development stage to increase accessibility, for example, a range of multimedia options (videos, audio, animations), limiting paragraph size, and using large font and high-contrast colours [43–45]. Developers may also consider incorporating clear instructions, training, or technical support into the intervention, as well as actively engaging people with low health literacy in the development of the interventions.

Developing interventions which are functional across multiple platforms may also increase accessibility. Whilst 20% of participants in study 1 did not have access to a smartphone or a tablet, it is possible that they do have access to a laptop or desktop computer and would therefore be able to benefit from an intervention delivered through a website. The participants in this study who did not have access to mobile devices were older. Evidence suggests these women may be more likely to have access to laptops or desktop computers than to mobile devices [46]. This was also supported by the results of the online study, as when participants were asked specifically about a potential digital intervention, there was a clear preference across all ages for interventions which are functional across both apps and websites. As reported preference for neither an online nor app-based intervention was low, combined estimates suggest that only 4–23% of breast cancer survivors would be reluctant to engage in an e-health intervention. Although this indicates that acceptability of an e-health intervention would be high across the ER + breast cancer population, it should be noted that women in study 2 were not asked specifically about their preference for a face-to-face or paper-based intervention.

There are limitations to this study which should be noted. Firstly, participants in study 1 were not asked about access to laptop or desktop computers, so it is not possible to ascertain access to these devices for the clinic sample. Only women who were fluent in English were eligible, so the results may miss participants who did not speak English, and this may have contributed towards the relatively small proportion of women from minority ethnic groups. There was a small proportion of women who did not provide data on the eHEALS scale, and these women were more likely to be older, from deprived areas and to be less educated. This suggests that the e-health literacy scores presented here may be overestimating the e-health literacy of the population. The percentage of women from minority ethnic groups was low, although this may be representative of the UK population of breast cancer survivors, as incidence of ER + breast cancer is lower in minority ethnic groups who are more likely to have triple-negative breast cancer than White women and are diagnosed at a younger age [46, 47].

## Conclusions

These findings have important implications, which are particularly salient in the current climate where the COVID-19 pandemic has led to a fast-tracked increase in digital health. These results provide support for e-health programmes in this population. However, there is still a digital divide which may cause disadvantage to some groups.

## Supplementary Information

Below is the link to the electronic supplementary material.

Supplementary file1 (DOCX 19 KB)

## Data Availability

Data will be made accessible upon reasonable request and in accordance with institutional review board data transfer guidelines.
