# Supplementary material for: Disparities in access to mobile devices and e-health literacy among breast cancer survivors
Source: Support Care Cancer. 2021 Jul 8;30(1):117–26. doi: 10.1007/s00520-021-06407-2 (PMC8264175; doi:10.1007/s00520-021-06407-2)
Supplement: Supplementary file 1 — Supplementary file1 (DOCX 19 KB) [file 520_2021_6407_MOESM1_ESM.docx]

Article title: Disparities in access to mobile devices and e-health literacy among breast cancer survivors.

Journal: Supportive Care in Cancer

Author names: Zoe Moon, Mira Zuchowski, Rona Moss-Morris, Myra S. Hunter, Sam Norton, Lyndsay D. Hughes

Corresponding author: Dr Lyndsay Hughes, email: [Lyndsay.hughes@kcl.ac.uk](mailto:Lyndsay.hughes@kcl.ac.uk)

**Online Resource 1**

Questions about app usage and preferred delivery of a support programme were not included in study 1. As the sample from study 2 was recruited entirely online, it was expected that technology use and online behaviour would be high. To offset this, estimates from studies 1 and 2 were combined to calculate estimates back-weighted to the NHS population to be more representative of all breast cancer survivors. Using the proportion of access to any mobile device from study 1 (Table 1), upper-bound estimates for health-app usage and intervention delivery preference were calculated, assuming that those without access to any mobile devices would have reported; i) never using health apps and; ii) a preference for neither an online or app-based intervention. The formulae for these calculations are shown below:

**Health-related App Usage**

The equation used to calculate the back-weighted proportion of health-related app usage is shown in equation 1, and age-stratified calculations in equations 2-4.

$$Backweighted proportion of health apps usage=\left( x\times\left( \frac{a}{y} \right) \right)+\left( 1\times\left( \frac{b}{y} \right) \right)$$

Where x = proportion never used health apps in study 2, a = number with any mobile device access in study 1, b = number with no access to mobile devices in study 1, and y = total sample in study 1

(1)

*Total Sample*

$$\left( .18\times\left( \frac{1612}{2009} \right) \right)+\left( 1\times\left( \frac{397}{2009} \right) \right)=.34$$

(2)

*Aged <45*

$$\left( .13\times\left( \frac{158}{163} \right) \right)+\left( 1\times\left( \frac{5}{163} \right) \right)=.16$$

(3)

*Aged 56+*

$$\left( .31\times\left( \frac{951}{1280} \right) \right)+\left( 1\times\left( \frac{329}{1280} \right) \right)=.49$$

(4)

**Preference for Neither Online nor App-based Intervention**

The equation used to calculate the back-weighted proportion of preference for neither an online nor app-based intervention is shown in equation 5, and the total-sample calculation in equation 6. As there were no age-related differences in reported preference in study 2, only the total sample estimate was calculated.

$$Backweighted proportion with preference for neither intervention=\left( z\times\left( \frac{a}{y} \right) \right)+\left( 1\times\left( \frac{b}{y} \right) \right)$$

Where z = proportion reporting preference for neither online nor app intervention in study 2, a = number with any mobile device access in study 1, b = number with no access to mobile devices in study 1, and y = total sample in study 1

(5)

*Total Sample*

$$\left( .04\times\left( \frac{1612}{2009} \right) \right)+\left( 1\times\left( \frac{397}{2009} \right) \right)=.23$$

(6)
